# Molecular Phylogenetic Characterization of *Anguina Tritici* (Steinbuch, 1799) Filipjev, 1936 (Rhabditida: Anguinidae) on Barley from Iraq

**DOI:** 10.2478/jofnem-2022-0040

**Published:** 2022-09-30

**Authors:** Elena Fanelli, Raied Abou Kubaa, Ali Kareem Al-Taee, Francesca De Luca

**Affiliations:** 1 Istituto per la Protezione Sostenibile delle Piante, Bari, Consiglio Nazionale delle Ricerche, Via Amendola 122, 70126 Bari, Italy; 2 Department of Plant Protection, College of Agriculture and Forestry, University of Mosul, Ninevah, Iraq

**Keywords:** 18S rRNA gene, *Anguina tritici*, barley, identification, ITS, phylogeny, systematics, molecular biology, interaction

## Abstract

During a nematological survey in Iraq, in the Bashika area, Ninevah province, an anguinid nematode population was isolated from galls of infected barley plants. The morphological characteristics indicated that the recovered species is identical to *Anguina tritici*. The barley population of *A. tritici* was molecularly characterized by sequencing two ribosomal regions (ITS and 18S rRNA genes), and their phylogenetic analyses revealed the newly generated sequences are in sister relation to corresponding sequences of *A. tritici* from wheat in the Bayesian tree, providing further evidence that the host plant can contribute to the separation of new isolates of plant parasitic nematodes.

*Anguina tritici* (Steinbuch, 1799) Filipjev, 1936 is a highly specialized nematode with a narrow host range. It is commonly called wheat seed gall nematode as multiplication only occurs on wheat or closely related plants. It is worldwide distributed in all wheat-growing areas (Ozberk et al., 2011; EPPO, 2015). The nematode is considered a low-level risk pest because it is easy to detect and control and has been eliminated from many grain-growing areas. It has caused yield losses up to 70% to *Secale cereale* L. and 50% to *Triticum aestivum* L. (Anwar et al., 2001). However, the adoption of modern seed cleaning methods has contributed to almost elimination of this species from commercial wheat production in developed countries (Nicol and Rivoal, 2008; CABI, 2014). But its occurrence is still reported from North Africa, India, Iran, Turkey, Iraq, Bulgaria, and very recently, has known as a re-emergence pest in Pakistan (Bonjar et al., 2004; Nicol and Rivoal, 2008; Kausar and Khan, 2009; Mohamedova and Piperkova, 2013; Mukhtar et al., 2018). In Iraq, it has already reported from wheat-growing areas (Rao, 1921; ­Al-Bedawi et al., 1977; Ami and Taher, 2013; Ami et al., 2019), and its infection was estimated around 22.9%, causing 30% crop losses (Al-Beldawi et al., 1977; Stephan and Antoon, 1990). In the last decades, the wheat seed nematode was also found in Iraq, infecting barley with an infection rate up to 90%, suggesting the occurrence of a tentative new biotype of the nematode (Al-Talib et al., 1986; Mustafa, 2009; Ami et al., 2019; Qassem et al., 2021). The present study aims to characterize the recently recovered population of *A. tritici* from barley fields in Iraq by sequencing the ribosomal ITS and the partial 18S rRNA genes.

## Materials and Methods

### Sampling and nematode extraction

During 2019, six barley ears of the local barley cultivar were collected from two infected fields located in Shakhan and Bashika, Ninevah province (northern Iraq). Infected plants showed basal swelling of the stem, leaf distortion, thin and deformed ears, and spindle-shaped galls. Green and mature galls from barely spikes were dissected in petri dishes containing distilled water under a stereomicroscope. Different stages of the nematode were picked up on slides containing a drop of lactophenol, and then, measurements of eggs, J2, females, and males were taken under a light microscope.

Infected seeds were left in sterile nuclease-free water for 2 hr and then crashed to release emerging juveniles. From the crashed Bashika sample, three 25 **m**l aliquots containing emerged juveniles were transferred into 1.5-ml sterile tubes and conserved at **-**20**°**C. Molecular identification was carried out at the laboratory of IPSP-CNR in Bari, Italy.

### DNA extraction, amplification, and sequencing

Ten individual specimens of the Bashika population were handpicked and placed on a glass slide in 3 **m**l of lysis buffer (10 mM Tris–HCl, pH 8.8, 50 mM KCl, 15 mM MgCl_2_, 0.1% Triton X100, 0.01% gelatine with 90 **m**g/ml proteinase K) and then cut into small pieces by using a sterilized syringe needle under a dissecting microscope (De Luca et al., 2004). The samples were incubated at 65**°**C for 1 hr and then at 95**°**C for 15 min to deactivate proteinase K. DNA purity and concentration were quantified using a NanoDrop**Ô** spectrophotometer (ThermoFisher Scientific, MA, USA) and then was used for conventional PCR.

The two ribosomal regions were amplified by using the following sets of primers: (i) 18S ext (5'-TTGATTACGTCCCTGCCCTTT-3') and 28S ext (5'-TTTCACTCGCCGTTACTAAGG-3') for the amplification of the ITS region (Vrain et al., 1992); (ii) and 18SnF (5'-TGGATAACTGTGGTAATTCTAGAGC-3') and 18SnR (5'-TTACGACTTTTGCCCGGTTC-3') for the partial amplification of 18S rRNA gene (Kanzaki and Futai, 2002).

The size of amplification products was determined by comparison with the molecular marker Ladder 100 (Fermentas, St. Leon-Rot, Germany) following electrophoresis of 10 **m**l on 1.5% agarose gel. Purified ITS and 18S rRNA gene fragments were cloned in a TA cloning vector (Invitrogen, MA, USA), and two clones from each molecular marker were sequenced by MWG Eurofins (Germany). The newly obtained sequences were submitted to the GenBank database with the following accession numbers: ON146309-ON146310 for the ITS region and ON146324 for the 18S rRNA gene.

### Phylogenetic analyses

Both ITS and 18 rRNA gene sequences obtained in this study were aligned with the corresponding ITS and 18S sequences (57 and six sequences, respectively) of anguinids deposited in the database. Sequence alignments were performed using MAFFT V. 7 software (Katoh et al., 2019) with default parameters and were manually edited using BioEdit (Hall, 1999) in order to remove the poorly aligned or ambiguous regions. Some species of the genus *Ditylenchus* Filipjev, 1936 were used as outgroup taxa (Subbotin et al., 2005; Mobasseri et al., 2017).

Phylogenetic analyses of both sequence datasets were based on Bayesian inference (BI) using MrBayes 3.1.2 (Ronquist and Huelsenbeck, 2003). The best-fit model of DNA evolution, a general time reversible model including among site heterogeneity (GTR **+** G **+** I), was selected using the Akaike information criterion (AIC) using JModelTest V.2.1.10 (Darriba et al., 2012) for both ITS and 18S datasets. The Markov chain Monte Carlo search was run with two default chains for 1,000,000 generations, sampled at every 100 trees. After discarding burn-in samples and evaluating convergence, the remaining samples were retained for in-depth analyses. Trees from all analyses were visualized using Dendroscope v3.2.8 (Huson and Scornavacca, 2012) and digitally drawn in CorelDRAW software version 2020.

## Results and Discussion

Close similarities were observed among juveniles and females collected from barley in the northern Iraq with *A. tritici* attacking wheat in Iraq (Ami and Taher, 2013). The infected barley fields showed typical swelling of the stem, deformation of leaves, thin and deformed ears, and spindle-shaped galls. The harvested seeds were also infected with the nematode, appearing darker, spotted on the surface, and smaller in size. Ami et al. (2019) already described the presence of three biotypes of *A. tritici* in three legume plants with high infection rates: (i) durum wheat biotype, (ii) the bread wheat biotype, and (iii) barley biotype. In the current study, nematodes collected from infected seeds were molecularly identified in order to investigate the affinities of the recovered tentative barley biotype.

The amplification of the ITS and 18S rRNA genes of this population yielded single fragments of 964 bp and 1,621 bp, respectively. Intraspecific variability of two ITS sequences of the barley population was 0.5% (5 mismatches). BLAST search at the NCBI revealed 95%–96% (36–47 mismatches and seven gaps) identity with the corresponding sequences of *A. tritici* available in the database.

**Figure 1:**
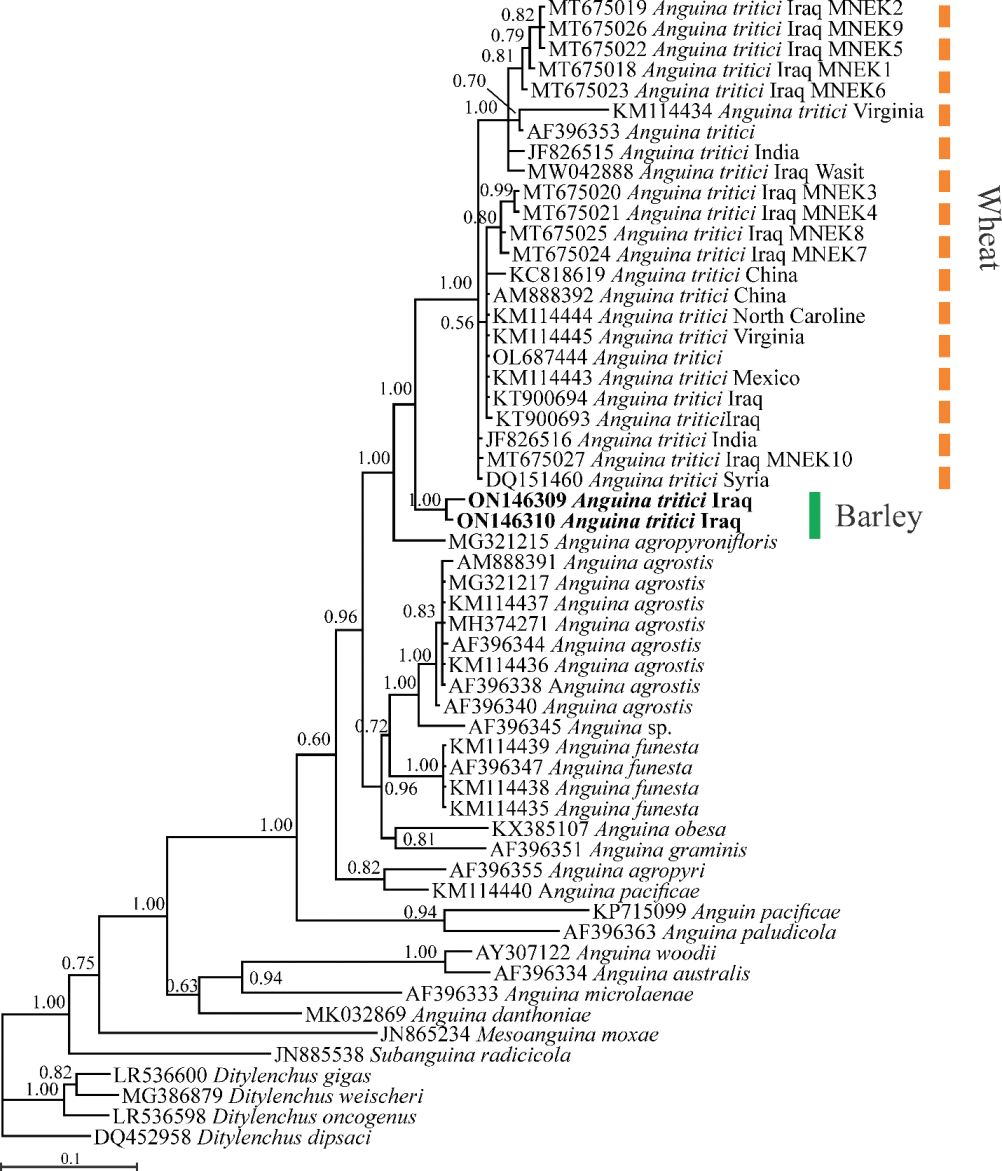
Bayesian phylogenetic tree of *Anguina tritici* (Steinbuch, 1799) Filipjev, 1936 from barley in Iraq, reconstructed using ITS rDNA sequences under the GTR **+** G **+** I model. Posterior probability values higher than 0.50 are given for appropriate clades. The newly obtained sequences are in bold font.

Phylogenetic relationships of *A. tritici* from barley from Iraq based on 57 ITS1-5.8S-ITS2 sequences are presented in Figure 1. In this tree, the newly generated sequences have formed a maximally supported clade with previously deposited sequences of *A. tritici* from wheat. *Anguina agropyronifloris* Norton, 1965 formed a clade with *A. tritici* sequences from wheat and barley, a similar relationship that was already resolved (Barrantes-Infante et al., 2018). The current phylogenetic analysis also confirmed the monophyly of *Anguina* and polyphyly of *Ditylenchus* (Aliverdi et al., 2021).

**Figure 2:**
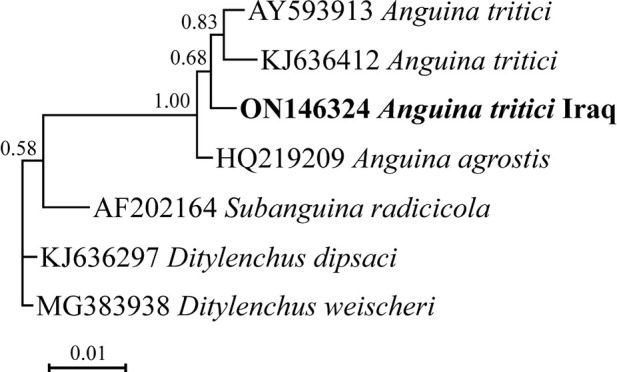
Bayesian phylogenetic tree of *Anguina tritici* (Steinbuch, 1799) Filipjev, 1936 from barley in Iraq, reconstructed using 18S rDNA sequences under the GTR **+** G **+** I model. Posterior probability values higher than 0.50 are given for appropriate clades. The newly obtained sequence is in bold font.

The phylogenetic tree reconstructed using 18S data is presented in Figure 2. In this tree, the newly generated sequence of *A. tritici* from barley has formed a clade with sequences of *A. tritici* from wheat currently available in GenBank.

The results of the current study confirm, for the first time, the existence of a barley biotype of *A. tritici* showing sister relationships with wheat biotypes using both ITS and 18S rRNA gene data. This finding could suggest that infection of different host plants can contribute to isolated nematode populations and has roles in their evolution (Powers et al., 2001; Subbotin et al., 2004; Mobasseri et al., 2017). These findings are in agreement with recent studies on *Meloidogyne incognita* (Kofoid and White, 1919) Chitwood, 1949 biotypes (Koutsovoulos et al., 2019) showing adaptative potential to different environments, geographical distribution, and host plants. This study revealed that *M. incognita* pathotypes do not share an evolutionary common ancestor; instead, it is likely that the same pathotypes have arisen independently multiple times in different regions. Furthermore, our results suggest the existence of *A. tritici* species complex, as reported in *Ditylenchus dipsaci* (Kühn, 1857) Filipjev 1936 (Yavuzaslanoglu et al., 2018; Storelli et al., 2021). Different populations of *D. dipsaci* showed variations in virulence and pathogenicity on sugar beet compared to onion, and only one of these populations/biotypes was highly adapted to sugar beet (Storelli et al., 2021). In conclusion, the two forms of *A. tritici* adapted to different host plants were genetically heterogenous and occupied different placements in presently resolved phylogenies.
